# Active Shape Modeling of the Hip in the Prediction of Incident Hip Fracture

**DOI:** 10.1002/jbmr.254

**Published:** 2010-09-27

**Authors:** Julie C Baker-LePain, Kali R Luker, John A Lynch, Neeta Parimi, Michael C Nevitt, Nancy E Lane

**Affiliations:** 1 Department of Medicine, University of California–San FranciscoSan Francisco, CA, USA; 2 Department of Orthopedic Surgery, Stanford University Medical SchoolStanford, CA, USA; 3 California Pacific Research InstituteSan Francisco, CA, USA; 4 Department of Medicine, University of California–DavisSacramento, CA, USA

**Keywords:** ACTIVE SHAPE MODELING, HIP SHAPE, HIP FRACTURE, OSTEOPOROSIS, BONE

## Abstract

The objective of this study was to evaluate right proximal femur shape as a risk factor for incident hip fracture using active shape modeling (ASM). A nested case-control study of white women 65 years of age and older enrolled in the Study of Osteoporotic Fractures (SOF) was performed. Subjects (*n* = 168) were randomly selected from study participants who experienced hip fracture during the follow-up period (mean 8.3 years). Controls (*n* = 231) had no fracture during follow-up. Subjects with baseline radiographic hip osteoarthritis were excluded. ASM of digitized right hip radiographs generated 10 independent modes of variation in proximal femur shape that together accounted for 95% of the variance in proximal femur shape. The association of ASM modes with incident hip fracture was analyzed by logistic regression. Together, the 10 ASM modes demonstrated good discrimination of incident hip fracture. In models controlling for age and body mass index (BMI), the area under receiver operating characteristic (*AUROC*) curve for hip shape was 0.813, 95% confidence interval (CI) 0.771–0.854 compared with models containing femoral neck bone mineral density (*AUROC* = 0.675, 95% CI 0.620–0.730), intertrochanteric bone mineral density (*AUROC* = 0.645, 95% CI 0.589–0.701), femoral neck length (*AUROC* = 0.631, 95% CI 0.573–0.690), or femoral neck width (*AUROC* = 0.633, 95% CI 0.574–0.691). The accuracy of fracture discrimination was improved by combining ASM modes with femoral neck bone mineral density (*AUROC* = 0.835, 95% CI 0.795–0.875) or with intertrochanteric bone mineral density (*AUROC* = 0.834, 95% CI 0.794–0.875). Hips with positive standard deviations of ASM mode 4 had the highest risk of incident hip fracture (odds ratio = 2.48, 95% CI 1.68–3.31, *p* < .001). We conclude that variations in the relative size of the femoral head and neck are important determinants of incident hip fracture. The addition of hip shape to fracture-prediction tools may improve the risk assessment for osteoporotic hip fractures. © 2011 American Society for Bone and Mineral Research.

## Introduction

Hip fracture is a major cause of morbidity and mortality for the elderly worldwide.(1) In the United States alone, it is estimated that 280,000 hip fractures occur annually.(2) The susceptibility of bone to fracture is determined by its biomechanical strength, which, in turn, is determined by the material of which bone is composed and the distribution and organization of this material.(3)

Among the many risk factors for hip fracture, low bone mineral density (BMD) and advanced age explain approximately 60% of the variance in the occurrence of this fracture type.(3) The ability to better predict incident hip fractures could lead to interventions that would prevent or delay the occurrence of such fractures and their significant associated morbidity. Recently, it has been demonstrated that measurements of proximal femur shape improve prediction models of hip fracture.(4) The shape of the proximal femur determines how mechanical forces are distributed during falls.(5) In a pilot study (*n* = 50), Gregory and colleagues found that the addition of proximal femur shape to BMD resulted in a 90% discriminatory accuracy of fracture prediction compared with 82% accuracy using BMD alone.(4) In another study, Beck and colleagues(6) reported that the strength of the proximal femur was better predicted by bone geometry than by BMD, highlighting the importance of hip shape in determining fracture risk.

The ability to determine proximal femur shape has been advanced by the development of active shape modeling (ASM). ASM is a technique for statistical modeling of shape, providing an average shape for the object being examined as well as principal modes of variation of that shape within the population of interest.(4) Since the introduction of this technique by Cootes and Taylor,(7) ASM has been applied widely in manufacturing,(8) facial recognition,(9) handwriting recognition,(10) and medical imaging of organs as varied as heart,(11) eye,(12) liver,(13) lung,(14) kidney,(15) and prostate.(16) Previously, we used ASM of the proximal femur to identify two modes of variation that were significantly associated with the incident radiographic hip osteoarthritis.(17) In this study, our goal was to evaluate the relationship between proximal femoral geometry, as assessed by ASM or other standard geometric measures such as femoral neck length or width, and incident hip fracture. We also determined whether the addition of hip shape to models of incident hip fracture improved the predictive accuracy of these models compared with BMD or other geometric measures.

## Methods

### Study subjects, case definition, and control selection

Cases and controls were sampled from the Study of Osteoporotic Fractures (SOF) database, a longitudinal multicenter cohort study of 9704 ambulatory white postmenopausal women recruited from population lists of four US cities.(18) Subjects were enrolled at age 65 years or greater between September 1986 and October 1988. In the current nested case-control study, cases consisted of 168 randomly selected postmenopausal women with incident femoral neck or intertrochanteric right hip fracture during the 8.3-year follow-up period. Presence of hip fracture was confirmed by medical record and radiologic review by a trained adjudicator. Controls consisted of 231 randomly selected postmenopausal women who did not experience hip fracture during the follow-up period. Subjects with radiographic hip osteoarthritis (RHOA) at baseline in either hip, defined by a summary score of 2 or more, modified from Croft,(19) were excluded owing to the likelihood that RHOA changes the femoral head shape. Additional exclusion criteria for this study included rheumatoid arthritis, Paget disease of bone, and history of previous hip fracture or of bilateral total hip replacements. The definition of RHOA has been described previously,(20,21) and radiographs were scored for baseline RHOA as described by Lane and colleagues.(22)

### Measurements

Height and weight at baseline visit were recorded using a wall-mounted Harpenden stadiometer (Holtan, Dyfed, UK) and balance beam scale, respectively. All participants completed a questionnaire at baseline that assessed self-reported health status, hours sedentary each day, education level, and current medication use.(17) Physical activity was assessed using a modified Paffenberger survey.(23) BMD of the femoral neck and total hip was measured at the baseline visit using dual-energy X-ray absorptiometry (DXA) with calibrated scanners (Hologic QDR 1000, Waltham, MA, USA). All scanners employed the same measurement program, and control phantoms were scanned daily to ensure proper calibration.(24)

### Radiographs

Subjects were assessed with supine anteroposterior pelvic radiographs at baseline and at follow-up visits using a standard protocol.(22) Radiographs of controls were obtained using the same X-ray equipment as for cases. A standard body position was used in all cases—supine with both legs extended and great toes touching one another, resulting in slight internal rotation of the femur. Radiographs were digitized with a VIDAR digitizer (VIDAR Systems Corp., Herndon, VA, USA) at a resolution of 0.169 mm (150 dpi) and stored as 16-bit DICOM images for further analysis.

Dimensions of the right proximal femur were measured using a digital image-analysis system (JiveX, Visus Technology Transfer, Bochum, Germany). Measurements of femoral head diameter, femoral neck length, and femoral neck width were done according to published methods.(25) Measurements of the proximal femur shape, femoral neck width, and femoral neck length were made by a single observer (KRL). Images were deemed unacceptable for analysis if the greater and lesser trochanters were not fully visualized; using this criterion, 8 of 176 cases (4.5%) were deemed unacceptable, leaving 168 images for ASM analysis.

### Active shape modeling (ASM)

ASM was performed using the method of Cootes and colleagues.(26) Digitized radiographs were evaluated by a reader (KRL), who outlined the shape of the femoral head and neck by placing a series of 60 evenly spaced points from the lesser trochanter to the opposite point on the femoral shaft.(17) The algorithms used were identical to those of Gregory and colleagues,(27) except for the number of points used (60 rather than 16) and inclusion of the entire proximal femur to the level of the lesser trochanter rather than only the femoral head and neck.(17)

Three hundred and ninety-nine baseline hip radiographs (168 cases, 231 controls) were entered into the ASM program to generate the composite average proximal femur shape of this sample, which formed the point of reference for comparison of variations from this average shape. The ASM program used principal components analysis (PCA) to calculate 10 independent “modes of variation” in hip shape, each of which independently described a portion of the overall variance in hip shape. Each of the 10 modes was independent, with no significant interactions between modes. Each individual hip was expressed in terms of standard deviations from the mean value of the 10 modes of variation. Together, the 10 modes of variation explained 95% of the total variance in proximal femur shape.(17)

### Statistical analysis

Statistical analyses were performed using STATA software (Version 11, Stata Corporation, Inc., College Station, TX, USA). Baseline subject characteristics were compared using Student's *t* tests and chi-square methods. *p* Values of .05 or less in two-tailed tests were considered significant. Each of the 10 modes of variation identified by ASM was included as an independent variable in a logistic regression model, with fracture case or control status as the outcome. Models were adjusted for baseline age, BMI, and femoral neck or intertrochanteric BMD as indicated.

Areas under the receiver operator characteristic curves (*AUROC*) were calculated to determine the probability that a randomly selected subject had an incident hip fracture based on a set of predictor variables including ASM modes and adjusting for age, hip BMD, and hip shape as indicated. World Health Organization (WHO) Fracture Risk Assessment Tool (FRAX) 10-year probabilites for hip fracture were calculated using an online calculator (http://www.sheffield.ac.uk/FRAX/tool.jsp) with variables from the SOF database.

To determine whether the association between hip shape ASM modes and incident hip fracture was due to chance alone, we performed an internal check by assigning hip fracture status randomly to each participant. Specifically, each individual subject was assigned a “case” or “control” status in the same proportion as the original analysis sample (1:1.38) or in a 1:1 or 1:2 case-to-control ratio.

## Results

### Baseline characteristics of the subjects

Subjects experiencing incident hip fracture during the follow-up period were slightly older and leaner than controls (age 71.7 ± 4.6 versus 70.6 ± 4.4 years, *p* = 0.01; BMI 25.5 ± 3.8 versus 26.6 ± 4.2 kg/m^2^, *p* = .02; Table 1). Hip fracture cases also reported less weight gain since the age of 25 years than did controls (8.6 ± 9.5 versus 11.4 ± 9.8 kg, *p* = .008). There was no significant difference between cases and controls with regard to exogenous estrogen use, vitamin D supplement use, smoking status, self-reported walking for exercise, or self-reported overall health status (Table 1). Fracture cases had significantly lower BMD of the total hip, femoral neck, and intertrochanteric region than controls (Table 1).

**Table 1:** Baseline Subject Characteristics^a^

	Cases (*n* = 168)	Controls (*n* = 231)	*p* Value
Age (years)	71.7 ± 4.6	70.6 ± 4.4	.01
Weight (kg)	65.1 ± 10.4	67.3 ± 10.9	.07
Height (cm)	159.9 ± 5.9	159.2 ± 5.9	.09
BMI (kg/m^2^)	25.5 ± 4.2	26.6 ± 4.2	.02
Estrogen use (%)	67 (39.9%)	96 (41.6%)	.74
Vitamin D use (%)	89 (53.3%)	127 (56.0%)	.60
Health status: good versus poor (%)	166 (98.8%)	229 (99.1%)	.75
Walks for exercise (%)	97 (57.7%)	112 (48.5%)	.07
Total-hip BMD (g/cm^2^)	0.70 ± 0.11	0.76 ± 0.12	<.0001
Femoral neck BMD (g/cm^2^)	0.60 ± 0.08	0.65 ± 0.10	<.0001
Intertrochanteric BMD (g/cm^2^)	0.82 ± 0.01	0.88 ± 0.01	<.0001
Femoral neck BMD *Z-*score^b^	−1.53 ± 0.60	−1.14 ± 0.70	<.0001

BMI = body mass index; BMD = bone mineral density.

a Values are mean ± SD unless otherwise indicated.

b Standardized to 65-year-old age group.

### Prediction models of incident hip fracture

We next examined the discriminative ability of various models for incident hip fracture. In these models of incident hip fracture, we included hip shape, site-specific BMD, femoral neck length, femoral neck width, or a combination of these variables as predictors (Table 2).

**Table 2:** *AUROC* Values for Various Models Predicting Hip Fracture, Adjusting for Age and BMI^a^

	Fracture site								
	
	All fractures (*n* = 168 fractures, 231 controls)			Femoral neck fractures (*n* = 86 fractures, 231 controls)			Intertrochanteric fractures (*n* = 75 fractures, 231 controls)		
			
Model	*AUROC*	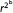	95% CI	*AUROC*	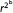	95% CI	*AUROC*	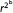	95% CI
Hip shape (modes 1–10)	0.813	0.239	0.771–0.854	0.832	0.259	0.786–0.878	0.824	0.237	0.771–0.877
Femoral neck BMD	0.675	0.063	0.627–0.730	0.683	0.065	0.616–0.749	0.666	0.051	0.595–0.737
Intertrochanteric BMD	0.645	0.045	0.589–0.701	0.640	0.042	0.572–0.708	0.639	0.037	0.565–0.712
Femoral neck length	0.631	0.039	0.573–0.690	0.675	0.078	0.607–0.743	0.612	0.029	0.533–0.691
Femoral neck width	0.633	0.041	0.574–0.691	0.625	0.077	0.566–0.684	0.613	0.019	0.554–0.673
Hip shape + femoral neck BMD	0.835	0.283	0.795–0.875	0.844	0.279	0.797–0.891	0.849	0.294	0.800–0.899
Hip shape + intertrochanteric BMD	0.834	0.277	0.794–0.875	0.837	0.268	0.789–0.885	0.843	0.280	0.793–0.894
Neck length + femoral neck BMD	0.691	0.077	0.633–0.748	0.705	0.095	0.636–0.774	0.695	0.071	0.622–0.768
Neck length + intertrochanteric BMD	0.664	0.058	0.605–0.723	0.671	0.071	0.600–0.743	0.670	0.054	0.595–0.746
Neck width + femoral neck BMD	0.680	0.064	0.621–0.739	0.671	0.083	0.611–0.730	0.669	0.051	0.609–0.729
Neck width + intertrochanteric BMD	0.655	0.051	0.594–0.715	0.639	0.069	0.578–0.700	0.645	0.041	0.584–0.706

*AUROC* = area under receiver operating characteristic curve; CI = confidence interval; BMD = bone mineral density; BMI = body mass index.

a Fractures are grouped as all fractures, femoral neck fractures, or intertrochanteric fractures. Controls for femoral neck fractures and intertrochanteric fractures were subjects with no fractures. Seven (*n* = 7) fractures were deemed neither purely femoral neck nor purely intertrochanteric and so were excluded from the site-specific fracture analysis.

b Pseudo-*r*^2^ value is presented.

Of the single predictors, ASM-derived hip shape demonstrated higher discriminative ability for incident hip fractures than the femoral neck or intertrochanteric BMD alone (*AUROC* = 0.813, 95% CI 0.771–0.854 for ASM versus 0.675, 95% CI 0.620–0.730 for femoral neck BMD versus 0.645, 95% CI 0.589–0.701 for intertrochanteric BMD). Femoral neck length and neck width had the lowest discriminative ability for hip fracture prediction after adjustment for age and BMI (*AUROC* = 0.631, 95% CI 0.573–0.690 for neck length; *AUROC* = 0.633, 95% CI 0.574–0.691 for neck width).

We next determined whether the combination of multiple predictors improved the accuracy of hip fracture-prediction models. In general, models including multiple predictors performed better than models with single predictors of hip fracture. For example, the best discriminatory ability was found in the model including the combination of both hip shape by ASM and femoral neck BMD (*AUROC* = 0.835, 95% CI 0.795–0.875).

Finally, we compared a hip fracture-prediction model including hip shape ASM modes with a model containing the 10-year probability of hip fracture as calculated by the WHO FRAX tool (http://www.sheffield.ac.uk/FRAX/tool.jsp). After adjusting for age and BMI, the AUROC for the model containing the 10 hip shape ASM modes was 0.813 (95% CI 0.771–0.854) compared with 0.651 (95% CI 0.586–0.715) for the model containing FRAX score. A model using only parental history of hip fracture had lower discriminatory power, with an *AUROC* of 0.602 (95% CI 0.538–0.665). Combining ASM modes with FRAX score produced a model with an *AUROC* of 0.806 (95% CI 0.756–0.856).

### Site-specific hip fracture-prediction models

We next evaluated site-specific hip fractures. For femoral neck fractures, the model that included hip shape ASM modes and femoral neck BMD had the highest discrimination of incident fractures (*AUROC* = 0.844, 95% CI 0.797–0.891). Femoral neck length or width contributed only minimally to improvement of the model for incident femoral neck fractures (Table 2). Hip shape by ASM showed the highest discrimination compared with femoral neck BMD, intertrochanteric BMD, femoral neck length, or femoral neck width (*AUROC* = 0.832, 95% CI 0.786–0.878 versus 0.683, 95% CI 0.616–0.749 versus 0.640, 95% CI 0.572–0.708 versus 0.675, 95% CI 0.607–0.743 versus 0.613, 95% CI 0.566–0.684, respectively).

For intertrochanteric fractures, the model including hip shape ASM modes and femoral neck BMD was the most accurate in predicting hip fracture (*AUROC* = 0.849, 95% CI 0.800–0.899). Hip shape by ASM showed better discrimination than femoral neck BMD, intertrochanteric BMD, femoral neck length, or femoral neck width (*AUROC* = 0.824, 95% CI 0.771–0.877 versus 0.666, 95% CI 0.595–0.737 versus 0.639, 95% CI 0.565–0.712 versus 0.612, 95% CI 0.533–0.691 versus 0.613, 95% CI 0.554–0.673, respectively). Interestingly, hip shape ASM modes explained an additional 10% to 20% of hip fracture discrimination not explained by BMD (Table 2).

### Fracture-prediction models according to strata of BMD

Since low BMD may confound the relationship between hip shape ASM modes and hip fracture, we stratified our sample population according to normal BMD (femoral neck BMD *T*-score > –1), osteopenia (femoral neck BMD *T*-score ≤ –1 but ≥ –2.5), and osteoporosis (femoral neck BMD *T*-score < –2.5) and examined the performance of hip fracture-prediction models. Hip shape ASM modes showed greater than 80% ability to correctly discriminate between fractures and nonfractures in all BMD strata, with the highest discriminatory ability seen in subjects with the lowest BMD scores (Table 3). Incorporation of femoral neck BMD into the model in addition to hip shape ASM modes improved discrimination in the normal BMD group and the osteopenic group, although this improvement was not statistically significant (Table 3). Addition of femoral neck BMD to the model along with hip shape ASM modes did not further improve hip fracture discrimination in the osteoporotic group (Table 3).

**Table 3:** *AUROC* Values for Hip Fracture According to Femoral Neck BMD *T*-Score Adjusted for Age and BMI^a^

	Normal BMD (*T* > –1.0, *n* = 44)			Osteopenic (–2.5 ≤ *T* ≤ –1.0, *n* = 236)			Osteoporotic (*T* < –2.5, *n* = 89)		
			
Model	*AUROC*	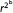	95% CI	*AUROC*	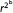	95% CI	*AUROC*	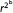	95% CI
Hip shape (modes 1–10)	0.813	0.239	0.771–0.854	0.818	0.245	0.764–0.872	0.862	0.343	0.784–0.939
Femoral neck BMD	0.675	0.063	0.620–0.730	0.661	0.061	0.592–0.732	0.574	0.009	0.449–0.700
Intertrochanteric BMD	0.645	0.045	0.589–0.701	0.606	0.031	0.534–0.679	0.547	0.004	0.423–0.670
Femoral neck length	0.631	0.039	0.573–0.690	0.647	0.061	0.572–0.722	0.623	0.012	0.496–0.750
Femoral neck width	0.609	0.018	0.550–0.668	0.630	0.049	0.572–0.689	0.458	0.003	0.397–0.519
Hip shape + femoral neck BMD	0.835	0.283	0.795–0.875	0.839	0.289	0.789–0.889	0.862	0.346	0.785–0.940
Hip shape + intertrochanteric BMD	0.834	0.277	0.794–0.875	0.827	0.259	0.774–0.879	0.868	0.362	0.792–0.944
Neck length + femoral neck BMD	0.691	0.077	0.633–0.748	0.694	0.094	0.622–0.766	0.647	0.016	0.520–0.773
Neck length + intertrochanteric BMD	0.664	0.058	0.605–0.723	0.649	0.061	0.574–0.724	0.623	0.012	0.495–0.750
Neck width + femoral neck BMD	0.391	0.112	0.329–0.453	0.683	0.075	0.625–0.742	0.345	0.010	0.285–0.405
Neck width + intertrochanteric BMD	0.502	0.159	0.438–0.566	0.641	0.050	0.581–0.701	0.465	0.003	0.401–0.528

BMD = bone mineral density; *AUROC* = area under receiver operating characteristic curve.

a *n* = 369 subjects had available BMD measurements. In the normal BMD group, 9 of 44 (20%) subjects were hip fracture cases; in the osteopenic group, 95 of 236 (40%) subjects were hip fracture cases; in the osteoporotic group, 53 of 89 (60%) subjects were hip fracture cases.

b Pseudo-*r*^2^ value is presented.

### Association of hip shape modes with hip fracture

**Fig. 1:**
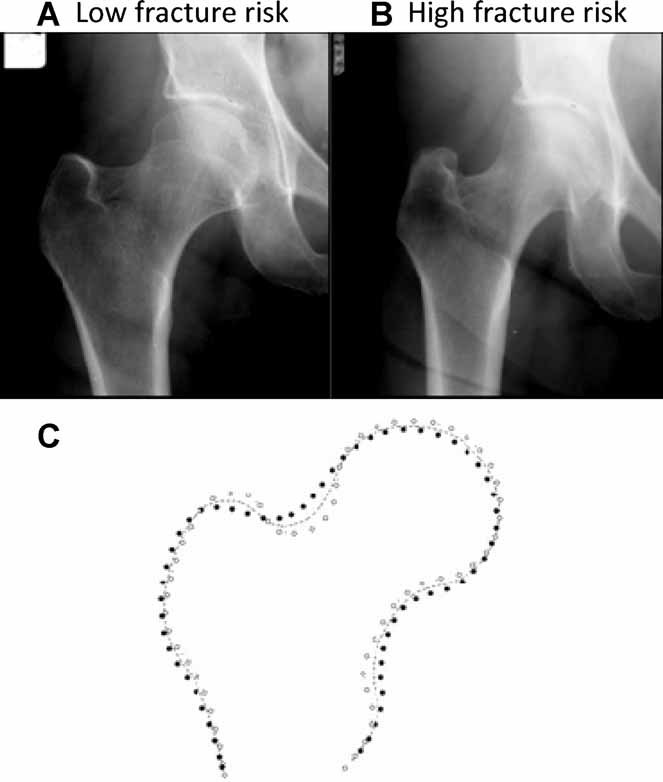
Representative radiographs depicting mode 4. *(A)* Hip with –0.3 standard deviations (SD) of mode 4. *(B)* Hip with +1.5 SD of Mode 4. Hips *(A)* and *(B)* are otherwise closely matched for Modes 1–3 and 5–10. *(C)* Cartoon showing mean shape of Mode 4 *(dashed line)* with ± 2 SD *(open and closed circles)* of this mode.

The ASM technique produced 10 independent modes of variation in hip shape by principal components analysis, which together explained more than 95% of the overall variation in hip shape in the population under study. In order to examine the relationship between individual ASM modes and incident hip fracture, we performed logistic regression modeling in which the 10 modes of variation were included as independent variables after adjusting for age, BMI, and femoral neck BMD (Table 4). Hips with more extreme values of mode 4 were associated with an increased risk of incident hip fracture (odds ratio [OR] = 2.48, 95% CI 1.68–3.31, *p* < .001). This mode was characterized by increased femoral neck length relative to a smaller femoral head size and a narrower femoral neck width and shaft width (Fig. 1). Hips with more extreme values of mode 5 also were associated with an increased risk of fracture, albeit of lesser magnitude than for hips high in mode 4 (OR = 1.32, 95% CI 1.06–1.66, *p* = .015). Modes 6, 8, and 10 appeared to confer protection from incident hip fracture (Table 4), but these modes explained little overall variance in hip shape compared with modes 1 to 5.

**Table 4:** Association Between Hip Shape Modes and Incident Hip Fractures After Adjusting for Age, BMI, and Femoral Neck BMD

	Odds ratio	95% CI	*p* Value
Mode 1	1.59	1.25–2.01	<.001
Mode 2	1.15	0.97–1.07	.22
Mode 3	0.89	0.71–1.11	.29
Mode 4	2.48	1.68–3.31	<.001
Mode 5	1.32	1.06–1.66	.015
Mode 6	0.56	0.44–0.72	<.001
Mode 7	1.19	0.95–1.48	.13
Mode 8	0.78	0.62–0.98	.03
Mode 9	0.95	0.76–1.18	.62
Mode 10	0.66	0.52–0.83	<.001

### Internal quality-control assessment

In order to examine whether the association of hip shape ASM modes with the risk of incident fracture was due to chance alone, we performed an additional logistic regression analysis assigning hip fracture status randomly to each participant. Specifically, each individual subject was assigned a “case” or “control” status in the same proportion as the original analysis sample (1:1.38). This resulted in 167 random cases and 232 random controls. In this analysis, there was no association between any of the hip shape ASM modes and case or control status. Inclusion of femoral neck BMD and/or femoral neck length or width did not result in a significant association between any hip shape ASM mode and case or control status. In order to confirm these results, we repeated the analysis using 1:1 and 1:2 case-to-control ratios, and again, we observed no significant association between hip shape ASM modes and case or control status, suggesting that our findings in the original case-control sample were not spurious.

## Discussion

In this community-based sample of postmenopausal white women, we found that hip shape as analyzed by ASM was a robust determinant of incident hip fractures. Furthermore, we found that hip shape performed better than BMD or FRAX scores in predicting incident hip fractures. The contribution of hip shape to prediction models of fracture is in concordance with several previous studies,(31–35) highlighting the idea that fracture likelihood depends on a combination of bone mass and bone geometry.(2) This study extends the results of previous findings by using a means of incorporating the shape of the entire proximal femur to the level of the lesser trochanter.

The combination of hip shape and BMD produced better discriminatory ability to predict future hip fractures than did single-predictor models. The need for incorporation of multiple variables in hip fracture-prediction models has been shown recently in studies reporting that the combination of bone shape, trabecular bone structure, and BMD performed better in predicting incident fracture than any of these variables used individually.(25,36,37) In current clinical practice, fracture-prediction tools such as the FRAX take into account individual characteristics (e.g., age, sex, ethnicity, height, and weight), past medical history and comorbidities (e.g., history of previous fracture or rheumatoid arthritis), family history (e.g., parent with history of hip fracture), environmental exposures (e.g., glucocorticoid use, smoking, and alcohol use), and BMD but do not currently include hip shape parameters.(38,39) Our results and those of others(31–35) suggest that incorporation of geometric measures of hip shape may improve the predictive accuracy of fracture-prediction tools. Indeed, when we compared models containing FRAX scores and those containing ASM with or without BMD, ASM performed better than FRAX in correctly predicting incident hip fractures in this population.

Which specific geometric parameters are most useful for fracture predictions is difficult to discern because these parameters have differed somewhat among various studies owing mainly to differences in measurement methodology. One advantage of the ASM method over other geometric measures is that it provides a relatively global mathematical description of the shape of an individual femur relative to the sample population rather than focusing on only one or two geometric measurements. Thus geometric features such as femoral neck-shaft angle or hip axis length are reflected in the ASM analysis, which in this case provided 10 modes of variation that described more than 95% of the overall variation in hip shape in the study population. Increased femoral neck-shaft angle, decreased cortical thickness, and increased hip axis length all have been found to be associated with increased fracture risk, with reported ORs of 1.4 to 2.0.(2,36,40,41) The effect of hip shape ASM mode 4 in this study conferred an increased OR of fracture of 2.48, which is roughly comparable with the magnitude of the effect of geometric measures seen in these other studies. In comparison with Gregory and colleagues,(25) who also used the ASM technique to evaluate incident hip fractures in the SOF population, our study incorporates nearly eight times as many subjects and had a more spatially detailed representation of the proximal femur shape (60 versus 29 points), allowing for greater statistical precision.

Although this study uniquely examines the discriminative ability of hip shape measurements using the ASM technique to predict future hip fractures, several limitations must be considered. First, the radiographic appearance of bone is influenced in part by the size of the patient. In our study, there was a significantly lower BMI in the fracture group than in the control group, although differences in height and weight did not reach statistical significance. To address this, we adjusted for BMI in final hip fracture-prediction models. Second, radiographic analysis is inherently limited in its ability to represent 3D objects in 2D images, creating the possibility that variations in hip rotation could bias the results. To minimize these effects, a standardized positioning was used for radiographs. In addition, Gregory and colleagues(25) found that the ASM technique is relatively robust to changes in both internal and external rotation in this cohort. Validation of our results using 3D techniques such as computed tomography (CT), however, will need to be performed. Third, our study did not incorporate the use of trabecular structure measurements, which have been added to some models incorporating ASM.(25) We did, however, incorporate bone density measurements, which provided some information about the trabecular structure of bone. Finally, the study population used here included only elderly white women. Thus the results of our analysis may not be generalizable to men, younger subjects, or other ethnic groups.

In summary, we have shown that variation in the relative shape of the femoral head is an important determinant of incident hip fracture. The combination of hip shape and femoral neck BMD provided the best discriminatory ability and may improve our ability to identify patients at increased risk for osteoporotic hip fracture. Since the number of fractures is projected to more than double in the next several decades, the importance of studying fracture prevention and treatment will have important implications for public health.(2)
